# Supplementary material for: Baicalein Inhibits Stx1 and 2 of EHE: Effects of Baicalein on the Cytotoxicity, Production, and Secretion of Shiga Toxins of Enterohaemorrhagic Escherichia coli
Source: Toxins (Basel). 2019 Aug 29;11(9):505. doi: 10.3390/toxins11090505 (PMC6784239; doi:10.3390/toxins11090505)
Supplement: Supplementary file 1 [file toxins-11-00505-s001.pdf]

Supplementary Materials: Baicalein Inhibits Stx1 and 2 of EHEC: Effects of Baicalein on the Cytotoxicity, Production and Secretion of Shiga Toxins of Enterohaemorrhagic *Escherichia coli*

Pham Thi Vinh, Yui Shinohara, Akifumi Yamada, Hoang Minh Duc, Motokazu Nakayama, Tadahiro Ozawa, Jun Sato, Yoshimitsu Masuda, Ken-Ichi Honjoh and Takahisa Miyamoto

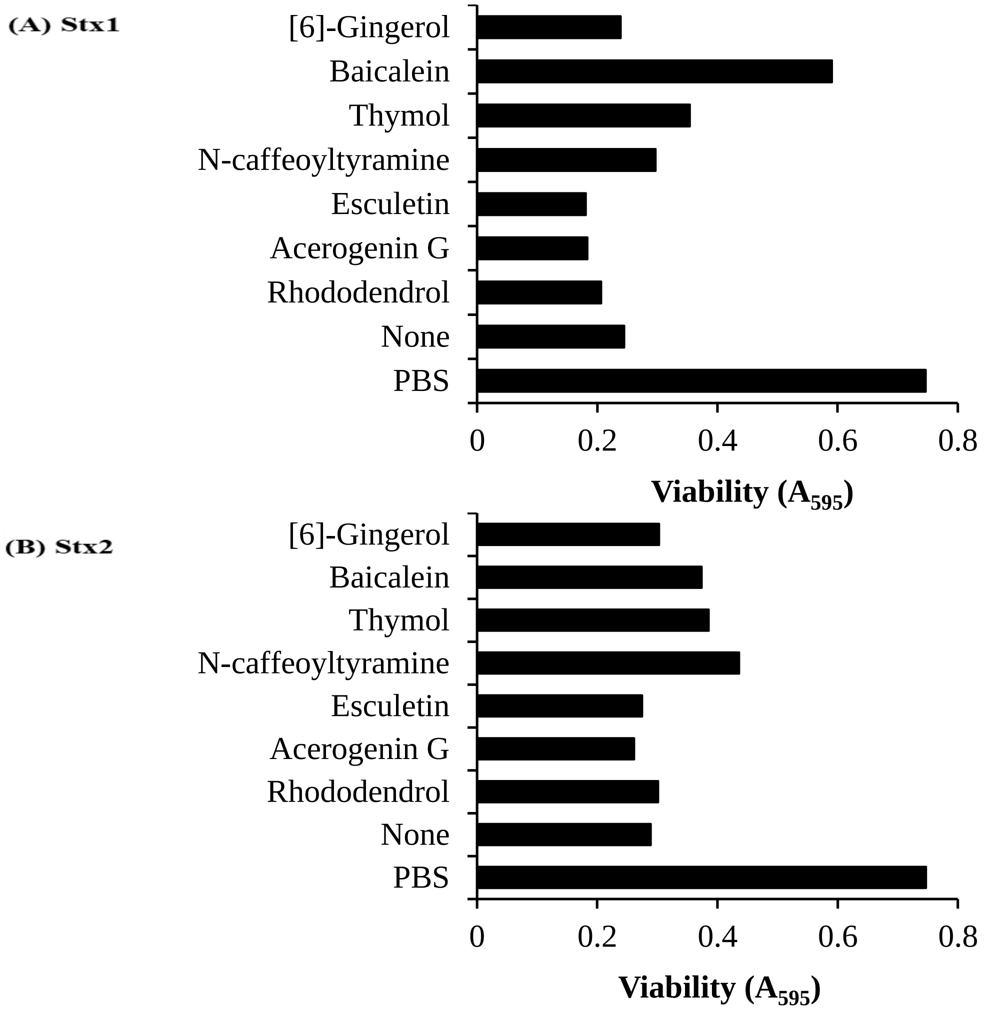

Figure S1. Effects of polyphenols on the cytotoxicity of Stx1 and Stx2. Stx1 (**A**) and Stx2 (**B**) preparations containing Stx1 and Stx2 at 12.5 and 50 mg/L, respectively were mixed without (None) or with 100 mg/L of each of the polyphenols and incubated incubated at 37 °C for 1 h. After the incubation, the mixture was added to the culture of Vero cells. Cell viability was determined by using MTT Cell Proliferation Assay after cultivation at 37 °C for 24. The values are the average of two separate experiments.
